# Spatiotemporal matching between medical resources and population ageing in China from 2008 to 2017

**DOI:** 10.1186/s12889-020-08976-z

**Published:** 2020-06-03

**Authors:** Junming Li, Xinglin Chen, Xiulan Han, Gehong Zhang

**Affiliations:** 1 School of Statistics, Shanxi University of Finance and Economics, Wucheng Road 696, Taiyuan, 030006 China; 2 First Hospital of Shanxi Medical University, Jiefang South Road 85, Taiyuan, 030001 China

**Keywords:** Population ageing, Medical resources, Matching measurement, Bayesian statistics

## Abstract

**Background:**

Globally, the increasingly severe population ageing issue has been creating challenges in terms of medical resource allocation and public health policies. The aim of this study is to address the space-time trends of the population-ageing rate (PAR), the number of medical resources per thousand residents (NMRTR) in mainland China in the past 10 years, and to investigate the spatial and temporal matching between the PAR and NMRTR in mainland China.

**Methods:**

The Bayesian space-time hierarchy model was employed to investigate the spatiotemporal variation of PAR and NMRTR in mainland China over the past 10 years. Subsequently, a Bayesian Geo-Detector model was developed to evaluate the spatial and temporal matching levels between PAR and NMRTR at national level. The matching odds ratio (OR) index proposed in this paper was applied to measure the matching levels between the two terms in each provincial area.

**Results:**

The Chinese spatial and temporal matching q-statistic values between the PAR and three vital types of NMRTR were all less than 0.45. Only the spatial matching Bayesian q-statistic values between the PAR and the number of beds in hospital reached 0.42 (95% credible interval: 0.37, 0.48) nationwide. Chongqing and Guizhou located in southwest China had the highest spatial and temporal matching ORs, respectively, between the PAR and the three types of NMRTR. The spatial pattern of the spatial and temporal matching ORs between the PAR and NMRTR in mainland China exhibited distinct geographical features, but the geographical structure of the spatial matching differed from that of the temporal matching between the PAR and NMRTR.

**Conclusion:**

The spatial and temporal matching degrees between the PAR and NMRTR in mainland China were generally very low. The provincial regions with high PAR largely experienced relatively low spatial matching levels between the PAR and NMRTR, and vice versa. The geographical pattern of the temporal matching between the PAR and NMRTR exhibited the feature of north-south differentiation.

## Background

The population aging problem has become increasingly severe throughout the world, with China as no exception [1, 2]. The average annual increase in the population-ageing rate (PAR) is 2.94% in mainland China, and, by the end of 2017, the number of citizens aged 65 and over reached nearly 160 million, accounting for 11.39% of the total population. Furthermore, data from the China country assessment report on aging and health 2015 (https://www.who.int/ageing/publications/china-country-assessment/en/) revealed that the deepening of the aging in China has increased the burden on society in terms of chronic diseases contracted by the elderly. Furthermore, the number of citizens affected by age-related diseases such as ischemic heart disease, cancer, arthritis and Alzheimer’s disease has gradually increased. By 2030, the number of elderly individuals suffering from one or more chronic diseases in China is projected to increase by at least 40% from 2020 [3].

The increasing PAR has been changing the structure of demographics and diseases, and therefore it has been changing the demand for medical care [4]. Some researchers have studied the impacts of population ageing on the medical resource (MR) of a whole country or region. Arai et al. [4] discussed the current situation regarding the impacts of population ageing from the perspective of available medical care resources in Japan as the front-runner of super-aged societies. The influence of population ageing on utilization rates of hospital services in Germany was studied by Schulz et al. [5] with a scenario simulation method. The data used in this research contained two types of datasets, one consisting of the main demographic data generated from the population forecasting model of the German Institute for Economic Research, and the other was the hospital diagnosis statistics data from the Federal Statistical Office of Germany and the study by Busse et al. [6]. This research concluded that changes in the number of ageing population will reorganise and restructure the allocation of the hospital departments. Based on surveys of outpatient hospitalization and the estimated population developed by the U.S. Bureau of the Census Population Projection Program, Strunk et al. [7] studied how the shift in the population age distribution of the USA will affect demand for hospital inpatient services. They found that population aging would drive about 0.74% annual growth in the use of medical services. Wang et al. [8] pointed out descriptively that inherent inequities existed in the healthcare insurance system in China and that the MRs were insufficient for the care of the elderly in China. Kwok et al. [9] employed a decomposition method to investigate the contribution of the ageing population to hospitalisation days in Hong Kong based on hospital discharge data between 2001 and 2012, and concluded that the rapid growth of the elderly population has made Hong Kong face an increasingly severe medical burden. The spatial distribution or equality of MR on a subnational or prefectural level was investigated in several previous studies. Based on the inventory or census of all public health facilities in the country, Rosero-Bixby [10] used the overlaying analysis of geographic information system (GIS) to evaluate the equity in access to healthcare for residents in Costa Rica at the subnational level. Liu et al. [11] and Zhang et al. [12] chose three indicators, number of institutions, number of health workers and number of beds, to study the equality of medical supply distribution in China on the subnational level from 2009 to 2013 and 2010–2014, using the relevant data from the China Health Statistics Yearbook. They concluded that the equity of per capita MR in China has been gradually improving; however, there has been a significant imbalance in terms of geographical distribution. The geographical accessibility and equality of medical supplies in Bhutan and Japan were analysed with the spatial accessibility indices and the Gini coefficient by Jamtsho et al. [13] and Shinjo and Aramaki [14] at the subnational scale. Research from Cheng et al. [15] and Wang et al. [16] utilized the nearest neighbour method to assess the spatial accessibility of healthcare in Beijing and the Sichuan Province at the prefectural level based on prefectural government statistics data and concluded that the spatial distribution patterns of health services in these two regions need to be further optimized. Additionally, several researchers have argued that successful ageing involves positive social support and a perfect healthcare system, and requires comprehensive consideration of MR supplies and corresponding fair access [17–20].

With regard to the relationship between PAR and MR, and although the impact of population ageing on the supply of MR was presented in previous studies, the analytical units in most of these studies were a whole country. Few discussed descriptively the relationship between PAR and MR in China. Some scholars have investigated the spatial pattern or accessibility of MR allocation at the subnational level. The data sources in these studies were mostly collected from corresponding government surveys or statistics, and the methods for detecting spatial accessibility or equality of MR were mainly ordinary spatial analysis, e.g. overlaying analysis, the nearest neighbour method, and the spatial Gini coefficient. To our knowledge, few studies have employed state-of-the-art spatiotemporal statistical methods to study the spatial and temporal variation of MR, and there is a lack of relevant research on the spatiotemporal matching between population ageing and MR in China and other countries at the subnational level. For some ageing populous countries, especially China, the allocation of MR needs to be reconstituted to respond to the increasing ageing population. The investigation of spatiotemporal matching of population ageing and MR can evaluate not only insufficient MR for the increasing ageing population, but also prospective policy-making references at the subnational level. Given the above information, our study adopted the state-of-the-art spatiotemporal statistical method, Bayesian space-time hierarchy model (BSTHM) [21], to investigate the spatial and temporal variation of the number of medical resources and PAR in mainland China over the past 10 years, from 2008 to 2017. The BSTHM integrating the Bayesian hierarchical model and space–time interaction model [21] can be used to model a coupled and complex space-time process by decomposing this into three sub-processes: overall spatial, common temporal, and space-time interaction effects [22]. Moreover, it is not possible to repeatedly sample in one spatial location at one time during a space-time process. The spatiotemporal data is not independent nor identically distributed (i.i.d.) due to the existence of spatiotemporal correlation [23]. In view of the above two points, we argue that classic statistical inference for space-time data is not reliable. However, the BSTHM can effectively overcome the problem of a small sample in the space-time process, and can make full use of spatiotemporal correlation as prior information. Based on the Bayesian spatiotemporal estimates of PAR and the number of medical resources per thousand residents (NMRTR), a Bayesian GeoDetector model was developed to quantify the spatiotemporal matching between PAR and the three types of NMRTR in mainland China at the national scale. In addition, a spatial and temporal matching odds ratio (OR) indicator proposed in this paper was used to analyse the spatial and temporal matching degree between PAR and NMRTR in each provincial region in mainland China.

## Methods

### Materials

The dataset used in this study was primarily comprised of two components, the first of which was the aged population and the total population of 31 provincial regions in mainland China from 2008 to 2017. This data was collected from the corresponding year’s Chinese Statistical Yearbook, and the population data referred to the population of permanent residents of the 31 provincial regions (http://www.stats.gov.cn/tjsj/ndsj/). The PAR in this paper refers to the proportion of the permanent population aged 65 years and over to the total permanent population. The second component was the MR of the same 31 provincial regions in mainland China from 2008 to 2017. The level of MR in each region was represented by medical personnel and facilities; among these, the medical personnel indicators include the number of licensed certified physicians per thousand residents (NLCPTR), and the number of registered nurses per thousand residents (NRNTR). The medical facilities indicators mainly include the number of beds in hospitals per thousand residents (NBHTR). Data on MR was collected from the Chinese Statistical Yearbook for the corresponding years.

### Bayesian space-time hierarchical model

The BSTHM proposed by Li and Haining et al. [21] in 2014, is a synthesis of the Bayesian hierarchical model and the space-time interaction model. It can disassemble the overall spatial relative magnitude and the local trends from a space-time coupling process. The mathematical expressions corresponding to the research questions in this paper were as follows:

1 $$ {Y}_{it}^{PAR}\sim N\left({\mu}_{it}^{PAR},{\sigma}_{PAR}^2\right)I\left(0,\right) $$

2 $$ \ln \left({\mu}_{it}^{PAR}\right)={\alpha}^{PAR}+{S}_i^{PAR}+\left({b}_0^{PAR}{t}^{\ast }+{v}_t^{PAR}\right)+{b}_i^{PAR}{t}^{\ast }+{\varepsilon}_{it}^{PAR} $$

3 $$ {Y}_{it}^{NMR}\sim N\left({\mu}_{it}^{NMR},{\sigma}_{NMR}^2\right)I\left(0,\right) $$

4 $$ \ln \left({\mu}_{it}^{NMR}\right)={\alpha}^{NMR}+{S}_i^{NMR}+\left({b}_0^{NMR}{t}^{\ast }+{v}_t^{NMR}\right)+{b}_i^{NMR}{t}^{\ast }+{\varepsilon}_{it}^{NMR} $$

where *t*^∗^ = *t* − 5 centering at the mid-observation period. ${Y}_{it}^{PAR}$ and ${Y}_{it}^{NMR}$ represent the PAR and three NMRTR data (NLCPTR, NRNTR, and NBHTR) of the *i*-th province in the *t* year. In consideration of ${Y}_{it}^{PAR}$ and ${Y}_{it}^{NMR}$ as continuous variables, they are assigned normal likelihood functions, and ${\mu}_{it}^{PAR}$, ${\mu}_{it}^{NMR}$, ${\sigma}_{PAR}^2$ and *σ*_2*NMR*_, are corresponding expectations and variances. *I*(0, ) denotes the range of greater than 0. Furthermore, *α*^*PAR*^ and *α*^*NMR*^ indicate the baseline of the PAR and NMRTR in mainland China during the study period; ${S}_i^{PAR}$ and ${S}_i^{NMR}$ represent the spatial relative risks of PAR and NMRTR during the study period, and $\exp \left({S}_i^{PAR}\right)$ and $\exp \left({S}_i^{NMR}\right)$ quantitatively measure the proportion of the PAR and NMRTR in the *i*-th provincial region relative to their corresponding national total levels, exp(*α*^*PAR*^) and exp(*α*^*NMR*^). If $\exp \left({S}_i^{PAR}\right)$ and $\exp \left({S}_i^{NMR}\right)$ are greater than 1.0, these indicate that the PAR and NMRTR in this region are $\exp \left({S}_i^{PAR}\right)$ and $\exp \left({S}_i^{NMR}\right)$ times the national total level and vice versa. $\left({b}_0^{PAR}t+{v}_t^{PAR}\right)$ indicates that the overall temporal trends of the PAR and NMRTR in mainland China; ${v}_t^{PAR}$ and ${v}_t^{NMR}$ represent the random variation effects of the overall temporal trends; ${b}_i^{PAR}$ and ${b}_i^{NMR}$ describe the local temporal trends of the PAR and NMRTR in the *i*-th provincial region from 2008 to 2017. The local temporal trends are stronger than the overall temporal trend, ${b}_0^{PAR}$ and ${b}_0^{NMR}$, if the parameters, ${b}_i^{PAR}$ and ${b}_i^{NMR}$, are greater than 0, and vice versa. ${\varepsilon}_{it}^{PAR}$ and ${\varepsilon}_{it}^{NMR}$ represent the random Gaussian noise in the process of space-time evolution.

Except for the observed data, $$ {Y}_{it}^{PAR} $$ and $$ {Y}_{it}^{NMR} $$, and the expectations of their normal likelihood distributions, $$ {\mu}_{it}^{PAR} $$ and $$ {\mu}_{it}^{NMR} $$, prior distributions were assigned for the other parameters of the BSTHM. $$ {S}_i^{PAR} $$, $$ {S}_i^{NMR} $$, $$ {b}_i^{PAR} $$, and $$ {b}_i^{NMR} $$, were assigned with the prior of the conditional auto regressive (CAR) model [24] integrated the Besag, York and Mollié (BYM) model [25]. The priors of the four parameters, *α*^*PAR*^, *α*^*NMR*^, $$ {b}_0^{PAR}, $$ and $$ {b}_0^{NMR} $$, were assigned non-informative priors. The reciprocals of $$ {\sigma}_{PAR}^2 $$ and *σ*_2*NMR*_ were assigned Gamma distributions. The Gaussian priors were assigned to the parameters, $$ {v}_t^{PAR} $$, $$ {v}_t^{NMR} $$, $$ {\varepsilon}_{it}^{PAR} $$ and $$ {\varepsilon}_{it}^{NMR} $$. According to the Bayesian statistical framework, all the parameters’ posterior distributions and their corresponding point estimations, e.g. means and medians, can be estimated based on the observed data, $$ {Y}_{it}^{PAR} $$ and $$ {Y}_{it}^{NMR} $$, and the assigned priors.

### Spatial and temporal matching OR index

According to the mathematical meaning of BSTHM, $\exp \left({S}_i^{PAR}\right)$ and $\exp \left({S}_i^{NMR}\right)$ represent the ratios of the overall stable PAR and one of the three types of NMRTR in the i-th provincial region relative to the total national level, exp(*α*^*PAR*^) and exp(*α*^*NMR*^). Consequently, the idea of OR index used in epidemiology can be used to construct the matching OR between the PAR and NMRTR. The mathematical expression was:

5 $$ {\mathrm{OR}}_{S_i}=\frac{\exp \left({\alpha}^{PAR}+{S}_i^{PAR}\right)/\exp \left({\alpha}^{PAR}\right)}{\exp \left({\alpha}^{NMR}+{S}_i^{NMR}\right)/\exp \left({\alpha}^{NMR}\right)}=\frac{\exp \left({S}_i^{PAR}\right)}{\exp \left({S}_i^{NMR}\right)} $$

According to the definition of the OR index, ${\mathrm{OR}}_{S_i}$ measures the OR between the PAR and a certain NMRTR in the *i*-th region, which can be regarded as a spatial matching OR. If ${\mathrm{OR}}_{S_i}$ > 1.0, this shows that a certain NMRTR does not matches spatially well with the PAR in the *i*-th region, which means that the proportion of a certain NMRTR in the *i*-th region to the overall national level is lower than the proportion of the PAR to the national level (and vice versa).

The above ${\mathrm{OR}}_{S_i}$ is a spatial matching metric. Similarly, $\exp \left({b}_i^{PAR}\right)$ and $\exp \left({b}_i^{NMR}\right)$ represent the ratios of the overall temporal trends of PAR and one of the three types of NMRTR in the *i*-th provincial region relative to the overall trends, $\exp \left({b}_0^{PAR}\right)$ and $\exp \left({b}_0^{NMR}\right)$. To measure the matching of dynamic change trends, the temporal matching OR index was expressed:

6 $$ {\mathrm{OR}}_{b_i,T}=\frac{{\left(\exp \left({b}_0^{PAR}+{b}_i^{PAR}\right)\right)}^T/\exp {\left({b}_0^{PAR}\right)}^T}{{\left(\exp \left({b}_0^{NMR}+{b}_i^{NMR}\right)\right)}^T/\exp {\left({b}_0^{NMR}\right)}^T}=\frac{{\left(\exp \left({b}_i^{PAR}\right)\right)}^T}{{\left(\exp \left({b}_i^{NMR}\right)\right)}^T} $$

T assigned with 5 years in this paper is duration of dynamic change. If ${\mathrm{OR}}_{b_i,T}$ > 1.0, this shows that the dynamic change of the PAR in the *i*-th area does not match temporally well with that of a certain NMRTR. This implies that the changing trend of a certain NMRTR in the *i*-th region dose not match that of the PAR (and vice versa).

### Bayesian geo-detector model

The Geo-Detector model [26, 27] is often used to analyse the spatial correlation of two variables. The basic idea of this approach is that if the spatial distribution of two variables is similar, the two variables can be considered strongly correlated. In other words, the q-statistic value calculated by the Geo-Detector model can measure similarities in the spatial distributions of two variables. A Bayesian Geo-Detector model was developed in this study. The mathematical expressions were as follows:

7 $$ {y}_i\sim N\left(\mu, {\sigma}^2\right) $$

8 $$ {y}_h\sim N\left({\mu}_h,{\sigma}_h^2\right) $$

9 $$ q=1-\frac{\sum \limits_{h=1}^l{N}_h{\sigma}_h^2}{N{\sigma}^2} $$

Where *q* is the q-statistic value measuring the matching degree between PAR and a certain type of NMRTR; h (1, 2, ...) represents the spatial stratification of a certain type of MR; *N*_*h*_ and *N* respectively represent the number of spatial units and the total number of spatial units contained in each layer; *μ*, *μ*_*h*_ and ${\sigma}_h^2$, *σ*^2^ respectively represent the mean and variance of the PAR within h and the total variance of the PAR in the study area. The priors of *μ* and *μ*_*h*_ were assigned non-informative prior distribution. The priors of $1/{\sigma}_h^2$ and 1/*σ*^2^ were assigned Gamma distribution. The posterior estimation of q-statistic value is between 0 and 1, and the larger the q-statistic value is, the more similar the spatial distributions of the number of a corresponding PAR and NMRTR. Based on the steady overall spatial pattern and local trends of the PAR and NMRTR estimated by the BSTHM, this paper calculated the spatial and temporal matching levels between the spatial distribution of the PAR and the thee NMRTR at national scale using the Bayesian Geo-Detector model. A large q-statistic value meant a similar spatial distribution or better matching between the PAR and a certain MR in the study area (and vice versa). In other words, the q-statistics index could be used as a direct measurement of the matching degree. All Bayesian statistical estimates in this paper were implemented using WinBUGS 1.4 software. The convergence of Markov chain Monte Carlo chains was assessed by the Gelman-Rubin index [28], which was below 1.05 for all parameters of the BSTHM.

## Results

### Descriptive statistics

In the past 10 years, the PAR and the three kinds of NMR in mainland China have exhibited a growth trend as well as clear regional differences. Figs. S1, S2, S3, S4 show the series of spatial and temporal distributions of the PAR and three NMRTR, (NLCPTR, NRNTR, and NBHTR), from 2008 to 2017. China’s PAR increased from 8.27% (Minmum: 6.61%, Maximum: 11.86%) in 2008 to 11.4% (Minmum: 6.94%, Maximum: 14.21%) in 2017. In 2017, except for Tibet, the PAR of other mainland Chinese provinces all exceeded the international limit of 7.00%. With the increase in the PAR, the burden of chronic diseases, such as hypertension, diabetes and cancer, will subsequently increase, while demand for MR, such as doctors, nurses, and hospital beds, will also continue to increase. In the past 10 years, the three types of MR in mainland China (e.g., NLCPTR, NRNTR, NBHTR) have also markedly increased, although significant spatial differences have emerged. The increase in the population of those over 65 years old has a rigid demand for nursing care. The three provinces with the highest NRNTR in 2017 were Beijing (4.77), Shanghai (3.47), and Zhejiang (3.32), while those with the lowest NRNTR were Anhui (2.21), Hebei (2.11), and Tibet (1.32). Except for Beijing, the NLCPTR, NRNTR in other provinces in 2017 was less than 4.00, which is lower than that of other more developed countries. In addition to medical facilities, the number of medical beds is also an important indicator of medical services. The NBHTR in mainland China has also greatly increased, however, the spatial pattern of the medical facilities is different from that of the medical personnel. In 2017, Liaoning (5.79), Xinjiang (5.37) and Qinghai (5.36) were the top three provinces with the highest NBH, while Tibet (3.49), Jiangxi (3.46) and Guangxi (3.31) exhibited the lowest.

### Spatiotemporal trends

#### Overall spatial trends

**Fig. 1:**
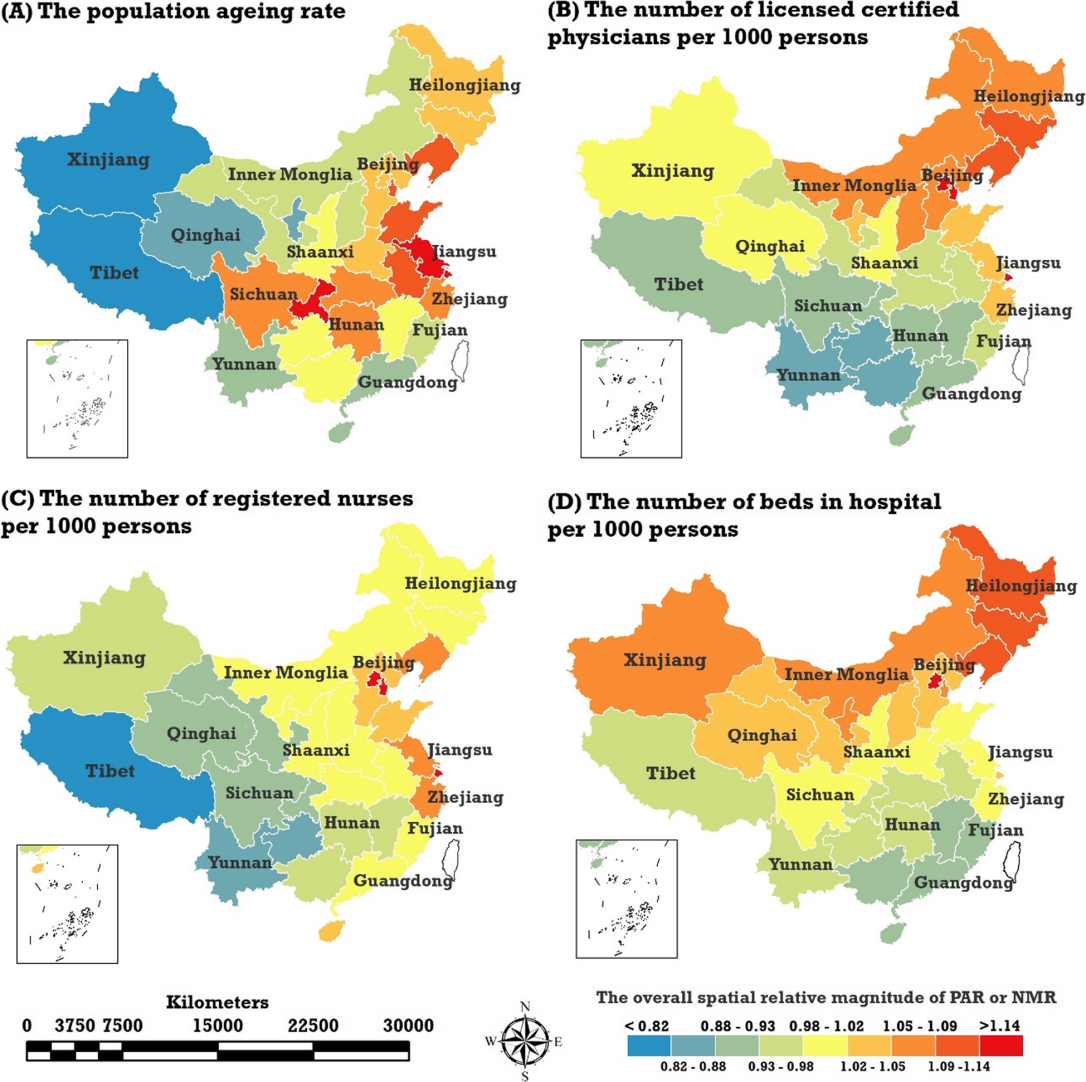
Overall spatial trends of China’s PAR (**a**) and three types of NMRTR: NLCPTR (**b**), NRNTR (**c**), NBHTR (**d**), the posterior medians of spatial relative risks, $\exp \left({S}_i^{PAR}\right)$ and the three types of $\exp \left({S}_i^{NMR}\right)$, estimated by the BSTHM. (Map generated with ArcGIS 10.3 by authors)

The overall spatial trends of PAR and NMRTR, the posterior medians of spatial relative risks, $\exp \left({S}_i^{PAR}\right)$ and $\mathit{\exp}\left({S}_i^{NMR}\right)$, were estimated by the BSTHM. Figure 1 shows the overall spatial trends of the PAR and the three kinds of NMRTR in mainland China. The results show that the spatial trends of PAR are quite different from those of NMRTR. Over the past 10 years, the level of PAR in western China has been lower, while in the southwest, central, southern, and eastern parts of China it has been comparatively higher. In terms of NMRTR, the overall spatial trends of the NLCPTR, and NBHTR were similar; that is, the northeast and eastern coastal regions were higher than other areas, while the overall spatial pattern of the NRNTR showed a clear gradient structure, eastern, central, and western were divided into three successively reduced levels.

#### Local temporal trends

**Fig. 2:**
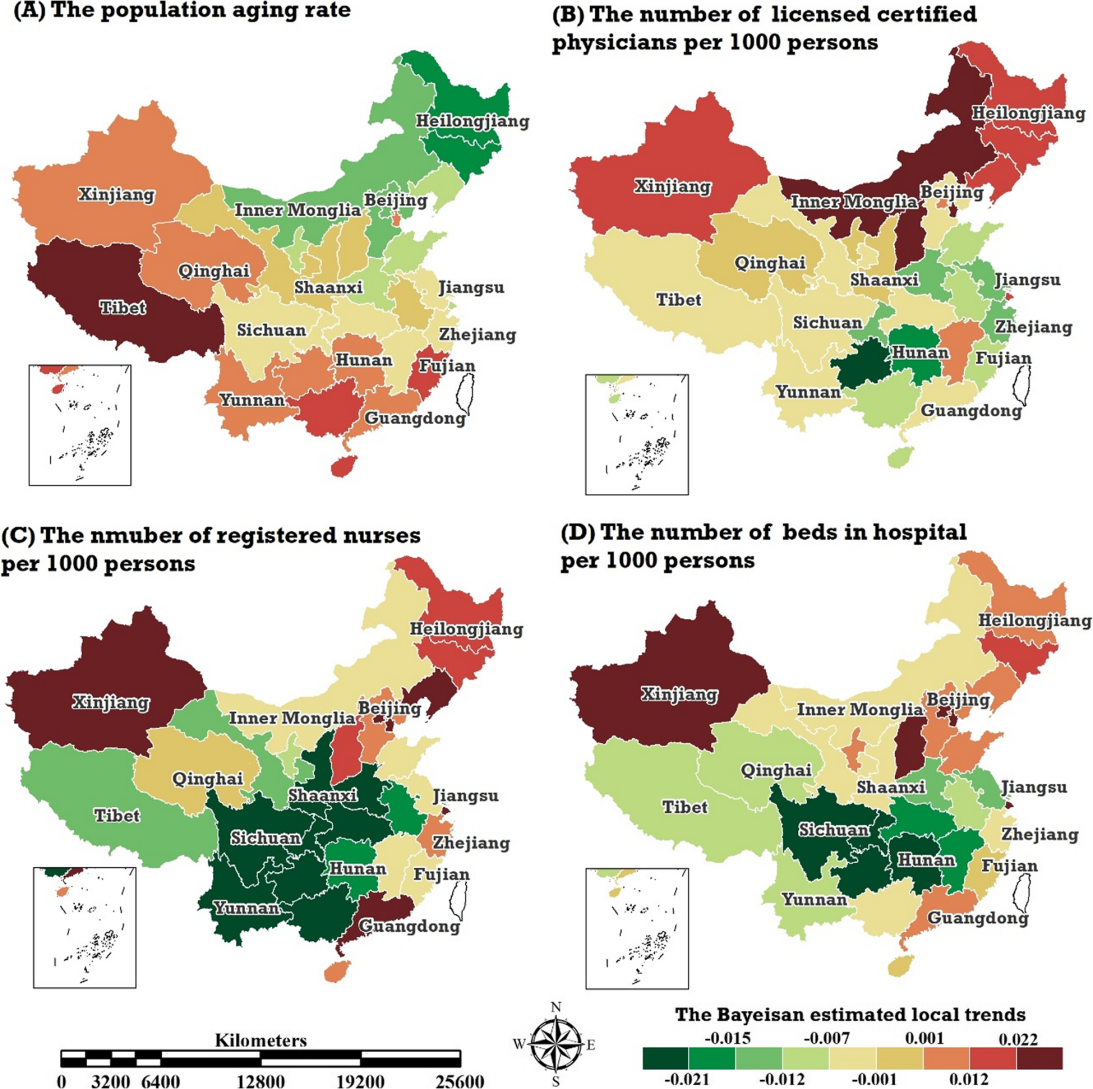
The spatial distribution of local temporal trends in China’s PAR (**a**) and three types of NMRTR: NLCPTR (**b**), NRNTR (**c**), NBHTR (**d**), the posterior medians of local temporal trends, ${b}_i^{PAR}$ and the three types of ${b}_i^{NMR}$, estimated by the BSTHM. (Map generated with ArcGIS 10.3 by authors)

Figure 2 shows the local trends of the PAR and the three kinds of NMRTR in each provincial region, the posterior medians of the parameters represent the local temporal trends, ${b}_i^{PAR}$ and ${b}_i^{NMR}$, and were estimated by the BSTHM. If the posterior medians of the local trend parameters, ${b}_i^{PAR}$ and ${b}_i^{NMR}$, were greater than 0, the local temporal trends of the PAR and NMRTR in the *i*-th provincial region would exhibit stronger increasing trends than the overall increasing trends, ${b}_0^{PAR}$ and ${b}_0^{NMR}$. Otherwise, if ${b}_i^{PAR}$ and ${b}_i^{NMR}$ were less than 0, the local temporal trends would exhibit weaker increasing trends relative to the overall increasing trends. From Fig. 2, it can be seen that the local trend of the PAR is quite different from that of the NMRTR. The local increasing trend of the PAR in the western and southern regions were stronger than that in the central and northern regions, and the regions with the strongest and weakest increase trends were Tibet and Heilongjiang, respectively. The spatial distribution of the local trend in NBHTR showed that northeast areas as well as eastern regions except Jiangsu presented a strong growth trend, while the south-central and southwestern regions revealed a weak growth trend. However, the spatial patterns of the local trends of the NLCPTR and NRNTR were all different. In terms of the NLCPTR, eight provincial regions located in the northern regions as well as Jiangxi, which is located in southeast China, exhibited a strong growth trend; meanwhile, a steady or weak increasing trend occurred in other areas. The NRNTR in 19 provinces exhibited a weak growth trend, and only six provinces (e.g., Xinjiang, Guangdong, Tianjin, Beijing, Shanghai, and Liaoning) exhibited a strong growth trend.

### Spatial and temporal matching between PAR and NMRTR

### Overall analysis

Table 1 listed the statistical results of Bayesian Geo-Detector q-statistic values for the spatial matching between the PAR and NMRTR. Only the q-statistic value between the NBHTR and PAR reached 0.42 (95% credible interval (CI): 0.37, 0.48) nationwide, while the q-statistic value ​​between PAR and NLCPTR, NRNTR, were 0.16 (95% CI: 0.12, 0.19) and 0.15 (95% CI: 0.12, 0.18). In other words, at present, except for the NBHTR, the matching degrees between the PAR and the other NMRTR were extremely low. This study analysed not only the spatial but also temporal matching degree of the PAR and NMRTR at the national scale (Table 1). Temporal matching refers to the matching level of the local trends between the PAR and NMRTR. At the national scale, the temporal matching degree between the PAR and NBHTR was slightly lower (q-statistic value was 0.37 (95% CI: 0.30, 0.44)) than the corresponding spatial matching degree. The other two types of NMRTR, NLCPTR and NRNTR, also exhibited a low temporal matching degree with the PAR, with corresponding q-statistic values of 0.22 (95% CI: 0.16, 0.28) and 0.17 (95% CI: 0.13, 0.21).

**Table 1:** Posterior median estimations and 95% CI of the Bayesian GeoDetector q-statistics values for the spatial and temporal matching between the PAR and the three types of NMRTR in mainland China

	The number of licensed certified physicians per thousand residents (NLCPTR) (95%CI)	The number of registered nurses per thousand residents (NRNTR) (95%CI)	The number of beds in hospital per thousand residents (NBHTR) (95%CI)
**Spatial**	0.16 (0.12, 0.19)	0.15 (0.12, 0.18)	0.42 (0.38, 0.49)
**Temporal**	0.22 (0.16, 0.28)	0.17 (0.13, 0.21)	0.37 (0.30, 0.44)

#### Provincial analysis

**Fig. 3:**
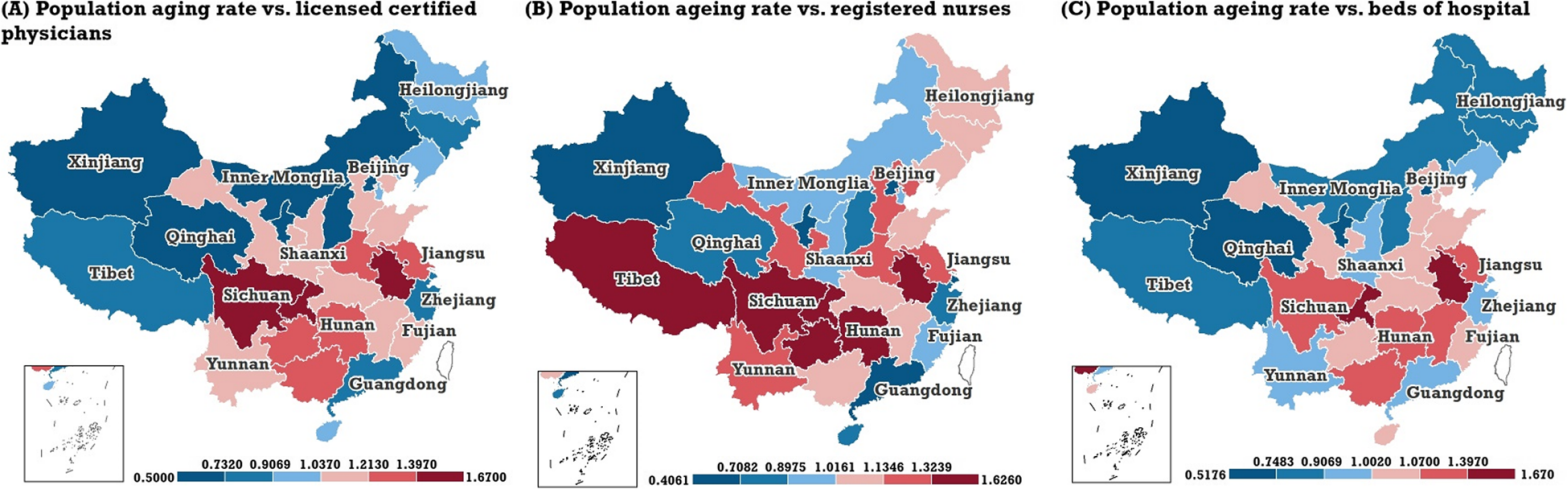
The spatial distribution of the spatial matching OR (Bayesian posterior median estimation) for mainland China’s PAR and three types of NMRTR: NLCPTR (**a**), NRNTR (**b**), and NBHTR (**c**). Spatial matching refers to the matching state based on the overall spatial trends. (Map generated with ArcGIS 10.3 by authors)

The spatial and temporal matching magnitude between the PAR and three important types of NMRTR—NLCPTR, NRNTR, and NBHTR at the provincial level can be quantified by the matching OR index proposed by this paper. Figure 3 and Table 2 show the Bayesian posterior medians and 95% CI of the spatial matching OR. The spatial matching ORs between the PAR and three types of NMRTR showed significant spatial differentiation. Generally, the spatial matching ORs between PAR and NLCPTR in western, northern, and south-eastern coastal areas (except for Fujian Province), were less than 1.0. The three provincial regions, Chongqing, Anhui, Sichuan, experienced the top three spatial matching ORs between the PAR and NLCPTR with 1.67 (95% CI: 1.61, 1.74), 1.59 (95% CI: 1.52, 1.65), and 1.40 (95% CI: 1.35, 1.45), respectively. The spatial matching degree between PAR and NRNTR in south-western and eastern coastal regions was weaker. The top four weakest matching degrees between the PAR and NRNTR occurred in four provincial regions, Chongqing, Sichuan, Anhui, and Tibet, and the corresponding spatial matching ORs were 1.63 (95% CI: 1.51, 1.76), 1.55 (95% CI: 1.45, 1.68), 1.50 (95% CI: 1.38, 1.62), 1.38 (95% CI: 1.21, 1.60), respectively. Furthermore, the spatial matching ORs in three provinces of northeast China were also greater than 1.0. The geographical pattern of the spatial matching OR between PAR and NBHTR (Fig. 3c) was generally similar to that between PAR and NLCPTR (Fig. 3a). The spatial matching ORs between PAR and NBHTR in western, northern, and southeast coastal areas (except for Fujian Province), were less than 1.0. Three provincial regions, Chongqing, Anhui, and Guangxi, experienced the top three spatial matching ORs, 1.45 (95% CI: 1.40, 1.50), 1.42 (95% CI: 1.37, 1.48), and 1.39 (95% CI:1.33, 1.45). Compared with the overall spatial pattern of the PAR (Fig. 1a), the provincial regions with high PAR have simultaneously weak matching degrees between the PAR and NMRTR, and vice versa. It needs to be noted that the PAR in Tibet was not high, but the matching degree between PAR and NRNTR was still weak; the corresponding spatial matching OR was 1.38 (95% CI: 1.21, 1.60). Additionally, although the PARs of three provinces in north-eastern China were not low, the spatial matching degrees between the PAR and NLCPTR, NBHTR, were not weak.

**Table 2:** The Bayesian posterior median and 95% CI of spatial matching OR between the PAR and three types of NMRTR in the 31 provincial regions in mainland China

Provincial regions	Spatial matching OR between PAR and NLCPTR (95% CI)	Spatial matching OR between PAR and NRNTR (95% CI)	Spatial matching OR between PAR and NBHTR (95% CI)
Beijing	0.50 (0.48, 0.52)	0.41 (0.39, 0.42)	0.72 (0.69, 0.74)
Tianjin	0.91 (0.87, 0.94)	0.93 (0.87, 0.98)	1.10 (1.06, 1.14)
Hebei	1.08 (1.04, 1.13)	1.31 (1.21, 1.42)	1.12 (1.08, 1.17)
Shanxi	0.72 (0.69, 0.75)	0.85 (0.79, 0.91)	0.84 (0.80, 0.88)
Inner Mongolia	0.73 (0.70, 0.76)	0.91 (0.85, 0.97)	0.88 (0.84, 0.92)
Liaoning	1.00 (0.97, 1.04)	1.03 (0.97, 1.09)	0.92 (0.89, 0.95)
Jilin	0.85 (0.82, 0.88)	1.03 (0.96, 1.10)	0.89 (0.86, 0.92)
Heilongjiang	0.97 (0.93, 1.01)	1.07 (1.00, 1.15)	0.86 (0.83, 0.90)
Shanghai	0.86 (0.83, 0.89)	0.64 (0.61, 0.67)	0.96 (0.93, 1.00)
Jiangsu	1.25 (1.20, 1.29)	1.20 (1.13, 1.27)	1.28 (1.24, 1.32)
Zhejiang	0.87 (0.84, 0.90)	0.86 (0.82, 0.91)	1.02 (0.99, 1.06)
Anhui	1.59 (1.52, 1.65)	1.50 (1.38, 1.62)	1.42 (1.37, 1.48)
Fujian	1.04 (0.99, 1.09)	0.95 (0.89, 1.02)	1.13 (1.08, 1.18)
Jiangxi	1.21 (1.16, 1.27)	1.13 (1.04, 1.23)	1.28 (1.23, 1.34)
Shandong	1.07 (1.03, 1.12)	1.08 (1.01, 1.15)	1.16 (1.11, 1.20)
Henan	1.24 (1.18, 1.30)	1.17 (1.08, 1.26)	1.05 (1.00, 1.10)
Hubei	1.14 (1.10, 1.18)	1.06 (1.00, 1.14)	1.16 (1.12, 1.20)
Hunan	1.38 (1.33, 1.43)	1.34 (1.24, 1.44)	1.25 (1.20, 1.29)
Guangdong	0.86 (0.82, 0.91)	0.70 (0.65, 0.75)	0.96 (0.91, 1.02)
Guangxi	1.26 (1.21, 1.32)	1.11 (1.03, 1.19)	1.39 (1.33, 1.45)
Hainan	0.98 (0.93, 1.03)	0.75 (0.70, 0.80)	1.06 (1.01, 1.11)
Chongqing	1.67 (1.61, 1.74)	1.63 (1.51, 1.76)	1.45 (1.40, 1.50)
Sichuan	1.40 (1.35, 1.45)	1.55 (1.45, 1.68)	1.36 (1.32, 1.41)
Guizhou	1.37 (1.31, 1.44)	1.33 (1.23, 1.46)	1.07 (1.03, 1.12)
Yunnan	1.15 (1.09, 1.21)	1.14 (1.04, 1.24)	0.92 (0.88, 0.96)
Tibet	0.83 (0.78, 0.88)	1.38 (1.21, 1.60)	0.84 (0.79, 0.90)
Shaanxi	1.10 (1.05, 1.14)	0.96 (0.90, 1.02)	0.96 (0.92, 0.99)
Gansu	1.16 (1.11, 1.22)	1.28 (1.19, 1.40)	1.04 (0.99, 1.08)
Qinghai	0.70 (0.66, 0.74)	0.76 (0.71, 0.82)	0.64 (0.61, 0.67)
Ningxia	0.65 (0.62, 0.68)	0.71 (0.66, 0.76)	0.62 (0.60, 0.66)
Xinjiang	0.65 (0.61, 0.68)	0.60 (0.56, 0.64)	0.52 (0.49, 0.54)

**Fig. 4:**
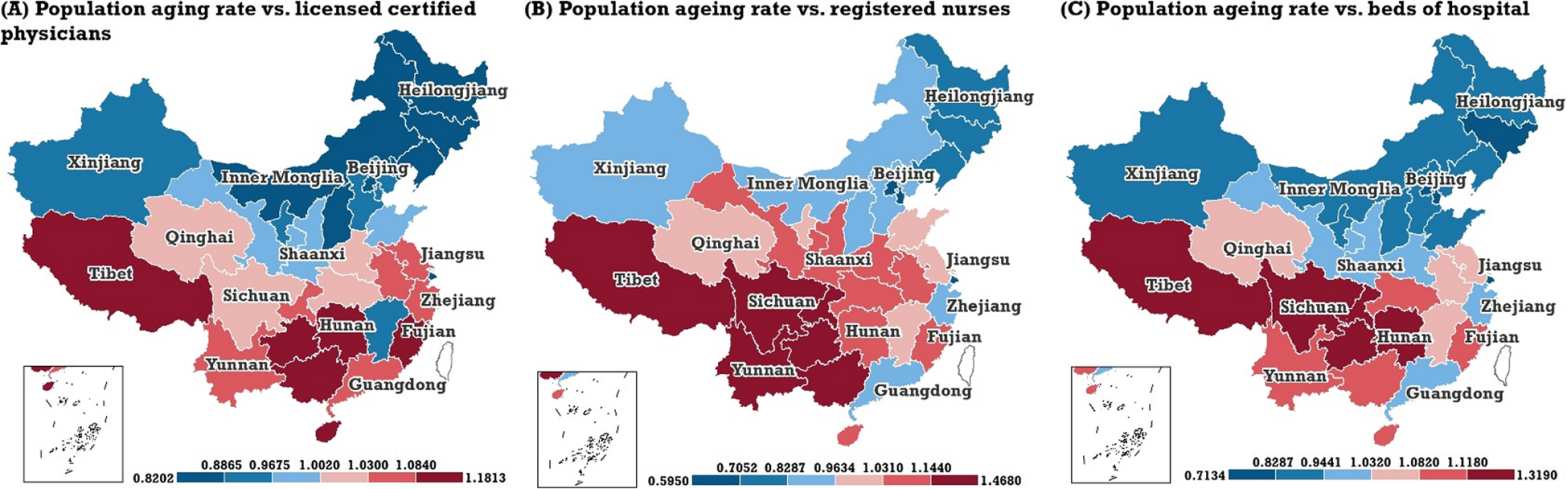
The spatial distribution of the temporal matching OR (Bayesian posterior median estimation) for mainland China’s PAR and three types of NMRTR: NLCPTR (**a**), NRNTR (**b**), and NBHTR (**c**). Temporal matching refers to the matching state based on the local temporal trends. (Map generated with ArcGIS 10.3 by authors)

From the view of the dynamic change trends, the Bayesian posterior medians and 95% CI of the temporal matching OR was estimated (Table 3) according to formula (6), and the temporal coefficient, T, was assigned as 5 years. In general, the spatial patterns of the temporal matching OR between the PAR and three types of NMRTR showed some similarities (Fig. 4). The temporal matching degree in the north was higher than that in the south. The three provinces with the largest temporal matching OR between the PAR and NLCPTR were Guizhou, 1.18 (95% CI: 1.10, 1.27), Tibet, 1.18 (95% CI: 1.07, 1.30), and Hainan1.15 (95% CI: 1.06, 1.26). The three provinces with the largest temporal matching OR between the PAR and NRNTR were Guizhou, 1.47 (95% CI: 1.29, 1.69), Yunnan 1.33 (95% CI: 1.17, 1.54), and Chongqing,1.26 (95% CI: 1.12, 1.41), while the three provinces with the largest temporal matching OR of the PAR and NBHTR were Guizhou,1.32 (95% CI: 1.24, 1.41), Tibet, 1.20 (95% CI: 1.10, 1.32), and Hunan, 1.18 (95% CI: 1.11, 1.25). Moreover, there are 10 northern provinces, including Heilongjiang, Jilin, Inner Mongolia, Shanxi, Beijing, Tianjin, Liaoning, Ningxia, Xinjiang, and Hebei, whose temporal matching ORs between the PAR and the three types of NMRTR were less than 1.0.

**Table 3:** The Bayesian posterior median and 95% CI of temporal matching OR between the PAR and three types of NMRTR in the 31 provincial regions in mainland China

Provincial regions	Temporal matching OR between PAR and NLCPTR (95% CI)	Temporal matching OR between PAR and NRNTR (95% CI)	Temporal matching OR between PAR and NBHTR (95% CI)
Beijing	0.89 (0.84, 0.94)	0.60 (0.56, 0.65)	0.71 (0.67, 0.76)
Tianjin	0.94 (0.88, 0.99)	0.71 (0.64, 0.78)	0.82 (0.77, 0.87)
Hebei	0.96 (0.89, 1.02)	0.92 (0.81, 1.05)	0.91 (0.85, 0.97)
Shanxi	0.88 (0.82, 0.94)	0.93 (0.83, 1.04)	0.87 (0.81, 0.94)
Inner Mongolia	0.83 (0.78, 0.89)	0.96 (0.86, 1.07)	0.94 (0.88, 1.01)
Liaoning	0.88 (0.83, 0.93)	0.82 (0.74, 0.91)	0.91 (0.86, 0.96)
Jilin	0.83 (0.78, 0.88)	0.83 (0.74, 0.92)	0.83 (0.77, 0.88)
Heilongjiang	0.82 (0.77, 0.88)	0.82 (0.73, 0.93)	0.87 (0.82, 0.93)
Shanghai	0.88 (0.82, 0.93)	0.60 (0.55, 0.64)	0.78 (0.74, 0.83)
Jiangsu	1.06 (1.01, 1.12)	1.01 (0.91, 1.12)	1.05 (1.00, 1.11)
Zhejiang	1.06 (1.00, 1.13)	0.95 (0.87, 1.04)	1.02 (0.95, 1.08)
Anhui	1.05 (0.98, 1.12)	1.08 (0.96, 1.22)	1.06 (0.99, 1.12)
Fujian	1.14 (1.06, 1.22)	1.10 (1.00, 1.23)	1.09 (1.02, 1.17)
Jiangxi	0.97 (0.90, 1.05)	1.01 (0.90, 1.16)	1.07 (0.99, 1.15)
Shandong	1.00 (0.94, 1.06)	0.99 (0.90, 1.10)	0.93 (0.88, 0.99)
Henan	1.03 (0.96, 1.11)	1.12 (0.99, 1.26)	1.03 (0.96, 1.10)
Hubei	1.03 (0.97, 1.09)	1.13 (1.02, 1.25)	1.10 (1.03, 1.17)
Hunan	1.11 (1.04, 1.19)	1.13 (1.01, 1.27)	1.18 (1.11, 1.25)
Guangdong	1.08 (1.00, 1.17)	0.92 (0.82, 1.02)	1.03 (0.95, 1.11)
Guangxi	1.12 (1.04, 1.20)	1.21 (1.09, 1.36)	1.11 (1.03, 1.19)
Hainan	1.15 (1.06, 1.26)	1.10 (0.98, 1.23)	1.12 (1.03, 1.21)
Chongqing	1.06 (0.99, 1.12)	1.26 (1.12, 1.41)	1.17 (1.11, 1.24)
Sichuan	1.02 (0.97, 1.08)	1.22 (1.09, 1.37)	1.17 (1.10, 1.23)
Guizhou	1.18 (1.10, 1.27)	1.47 (1.29, 1.69)	1.32 (1.24, 1.41)
Yunnan	1.08 (1.01, 1.18)	1.33 (1.17, 1.54)	1.12 (1.04, 1.20)
Tibet	1.18 (1.07, 1.30)	1.21 (0.99, 1.47)	1.20 (1.10, 1.32)
Shaanxi	1.00 (0.94, 1.07)	1.14 (1.04, 1.27)	1.03 (0.97, 1.10)
Gansu	1.00 (0.93, 1.08)	1.07 (0.94, 1.20)	1.00 (0.93, 1.07)
Qinghai	1.02 (0.94, 1.11)	1.03 (0.91, 1.17)	1.08 (1.00, 1.17)
Ningxia	0.96 (0.88, 1.04)	1.01 (0.90, 1.15)	0.91 (0.84, 0.99)
Xinjiang	0.96 (0.88, 1.04)	0.94 (0.84, 1.05)	0.89 (0.82, 0.96)

## Discussion

Population ageing is a severe challenge that every country in the world faces [1] . With the advent of population ageing, the composition of disease has changed dramatically in various countries around the world, which, subsequently, has led to a shift from traditional infectious diseases to chronic diseases (e.g., hypertension, heart disease, diabetes, etc.) [29]. The demand for medical services has also changed—specifically, increasing [9]. As a developing country with a large population base, China has become an aging country [30], experiencing a significant increase not only in the PAR but also the aging population. Furthermore, an increasing demand for MR is one of the challenges the Chinese government and other populous countries, currently have to face, a burden shared with other developing countries.

This paper first analysed the spatial and temporal process of the PAR and three types of NMRTR in mainland China in the past 10 years (2008–2017) by employing the BSTHM and estimated the results of their steady-state spatial patterns and dynamic local trends. On this basis, this paper also investigated the spatial and temporal matching degree between the PAR and three types of NMRTR in mainland China using the Bayesian Geo-Detector model and the matching OR index proposed in this paper. The results showed that there was an incongruency between the PAR and three types of NMRTR. The statistical results of the Bayesian Geo-Detector model revealed that the national spatial and temporal matching degrees were generally low. The relatively strong matching NMRTR was NBHTR, however, the corresponding matching q-statistic value was merely 0.42 (95% CI: 0.38, 0.49), less than 0.50. Among the three types of NMRTR, NLCPTR, NRNTR, and NBHTR, the spatial and temporal matching degrees of NBHTR were higher than other two types of NMRTR. .

Inspiring the idea of the OR index in epidemiology, this paper proposed a matching OR index based on the results estimated from the BSTHM. The matching OR index was used to assess the spatial and temporal matching degrees between the PAR and three typical NMRTR in mainland China. In terms of spatial matching, there generally existed a phenomenon that the areas with higher PAR simultaneously experienced weak matching with NMRTR, e.g. Sichuan, Anhui, and Chongqing. Except for NRNTR, the westernmost three provincial regions, Xinjiang, Tibet, Qinghai, whose PAR was the lowest over mainland China, had relatively strong matching degrees between PAR and NLCPTR, NBHTR. In view of NRNTR, more provincial areas possessed weak matching with PAR, including Tibet and the three provinces located in northeast China. In other words, the MR of nursing in China needs to be expanded under the aging background. The geographical features of temporal matching between the PAR and NMRTR differ from that of spatial matching. Xinjiang was the only region among the three westernmost three provincial regions with low PAR and relatively high temporal matching. Moreover, a distinct geographical structure with north-south differentiation occurred in the temporal matching between PAR and the three types of NMRTR in mainland China. The southwest regions of China, e.g. Tibet, Yunnan, Sichuan, Guizhou, and Guangxi, experienced very weak temporal matching between the PAR and the three classical NMRTR (Fig. 4).

As a large country with a vast territory, China has unbalanced characteristics in PAR and NMRTR. Therefore, when formulating public policies, it is necessary for the Chinese government to make scientific and precise policy decisions with full consideration of this spatial difference. Some interesting findings were obtained, which might aid the Chinese government in its formulation of relevant policies in the future and also provide some reference for other developing countries in the world.

There were still some shortcomings of this study to discuss. First, the provincial-level analysis unit is too big to measure accurately the accessibility of healthcare resources for the older population. In other words, the ratio in a provincial region can only roughly represent the accessibility of MR and the level of population ageing. Even so, the 31 provincial level regions in mainland China served as analytical units on account of the availability of data. Additionally, the statistical results at the provincial level can also provide some useful references for related national-level policy-making. Furthermore, in China, the provincial administrative government is the secondary administrative unit, and the Chinese central government’s fiscal expenditure of public health services is allocated by provincial regions (http://www.gov.cn/zhengce/content/2018-08/13/content_5313489.htm). Second, there existed the modifiable areal unit problem (MAUP) in our study due to the differences in the acreage of various provincial regions. We argued that the data used in our study was statistical yearbook data rather than geographical lattice data, e.g. remote sensing data. The PAR and NMR collected from the statistical yearbook data were determined by the total population and MR of a provincial region. Hence, PAR and NMRTR were not strongly associated with the acreage of provincial regions. Consequently, the MAUP may be avoided to some degree. In addition, the MAUP will still exist when the analysis is conducted at the sub-provincial level. Third, as mentioned above, the age-related diseases include ischemic heart disease, cancer, arthritis and Alzheimer’s, but the MRs in this study are the general physician, nurse, and bed. It will be better if the NMR is related to the ageing population, especially for the number of physicians. However, the corresponding and refined data could not be obtained. This is also a future study direction. In this paper, we assumed that the proportion of gerontologists relative to general physicians per 1000 persons in various provincial regions was comparable. Fourth, the population data in this paper that was collected from the statistical yearbook refers to permanent residents, but the census survey cannot reach the people without household accounting for almost 1%. Consequently, we assumed that the proportion of people without a household in every provincial region was approximate. Fifth, our study did not begin with the absolute quantity of ageing population’s demand for MR but instead from the perspective of a relative relationship, which needs to be further explored.

## Conclusions

The overall spatial patterns of China’s PAR and NMRTR showed distinct geographical differences. The PAR was high in the east and low in the west. The northeast and northern regions of China experienced higher level of the NMRTR than that in the Southern and Western regions. The increasing trend of the PAR in the west and south was fast, but slow in the northeast and north. The NMRTR experienced the stronger increasing local trend in the Northeast, North China, and Xinjiang than that in the Southwest and Southern regions. Generally, the matching degree between the PAR and NMRTR in mainland China was very low. The national spatial and temporal matching degrees of the PAR and NBHTR were higher than those between the PAR and NLCPTR, NRNTR. Except for NRNTR, the NLCPTR and NBHTR in the three westernmost and three northeast provincial regions matched better than that other areas. In this regard, the spatial patterns of the matching OR between the PAR and NMRTR in mainland China show clear geographical differentiation characteristics. The provincial regions with high PAR experienced a relatively low spatial matching degree between the PAR and NMRTR. The geographical pattern of the temporal matching between the PAR and NMRTR exhibited north-south differentiation.

## Supplementary information

**Additional file 1: **The population ageing ratio (**Figure S1**), number of licensed physicians per 10,000 persons (**Figure S2**), number of registered nurses per 10,000 persons (**Figure S3**), number of beds in hospital per 10,000 persons (**Figure S4**), in 31 provincial regions in mainland China from 2008 to 2017

## Data Availability

The spatiotemporal datasets that support the findings of this study are available in the corresponding year’s Chinese Statistical Yearbook, hyperlink to datasets source “http://www.stats.gov.cn/tjsj/ndsj/”.

## Abbreviations

MR: Medical resources
BSTHM: Bayesian space-time hierarchy model
OR: Odds ratio
PAR: Population ageing rate
NMRTR: Number of medical resources per thousand residents
NLCPTR: Number of licensed certified physicians per thousand residents
NRNTR: Number of registered nurses per thousand residents
NBHTR: Number of beds in hospitals per thousand residents
