# Supplementary material for: Spatiotemporal matching between medical resources and population ageing in China from 2008 to 2017
Source: BMC Public Health. 2020 Jun 3;20:845. doi: 10.1186/s12889-020-08976-z (PMC7268461; doi:10.1186/s12889-020-08976-z)
Supplement: Supplementary file 1 — Additional file 1: The population ageing ratio (Figure S1), number of licensed physicians per 10,000 persons (Figure S2), number of registered nurses per 10,000 persons (Figure S3), number of beds in hospital per 10,000 persons (Figure S4), in 31 provincial regions in mainland China from 2008 to 2017 [file 12889_2020_8976_MOESM1_ESM.docx]

**Spatiotemporal Matching between Medical Resources and Population Ageing in China from 2008 to 2017: Supplementary Materials**


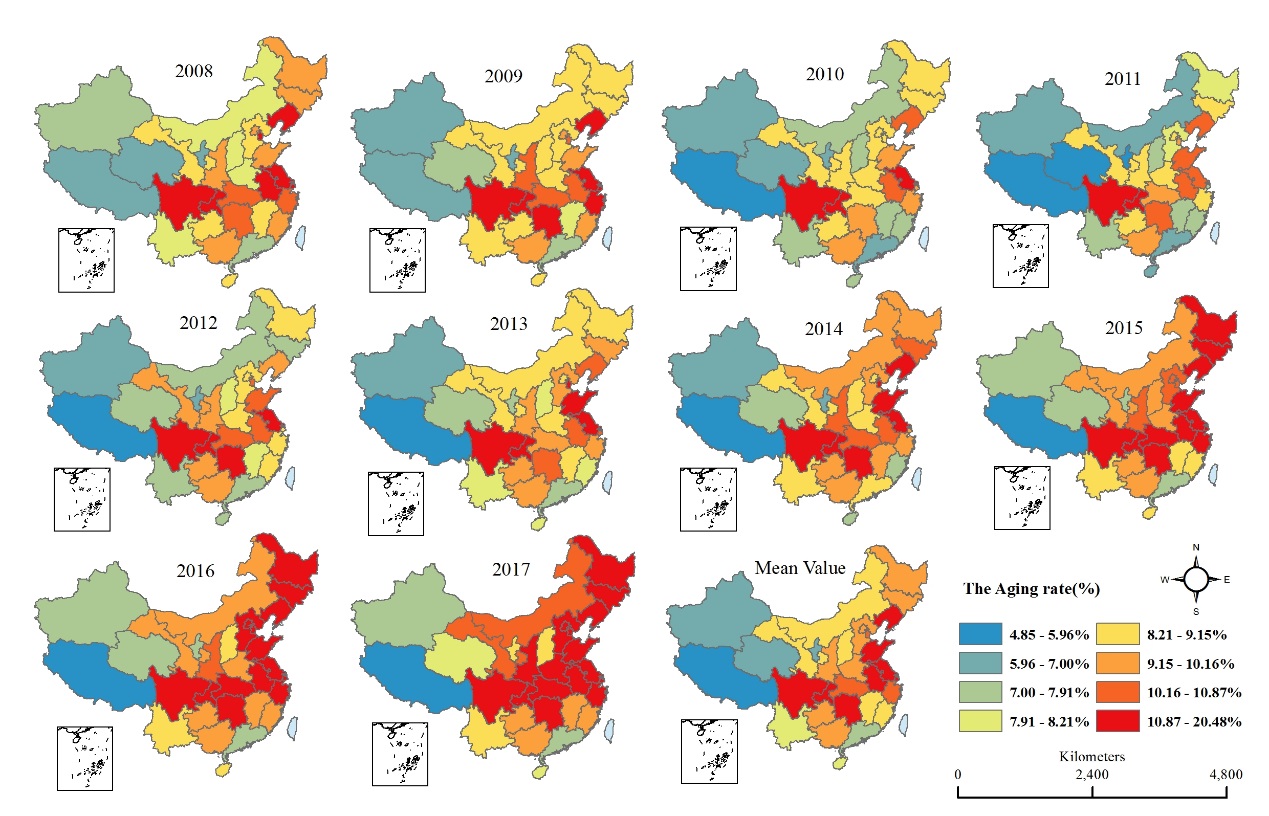


Figure S1. The population ageing ratio in 31 provincial regions from 2008 to 2017 in mainland China (Map generated with ArcGIS 10.3 by authors)


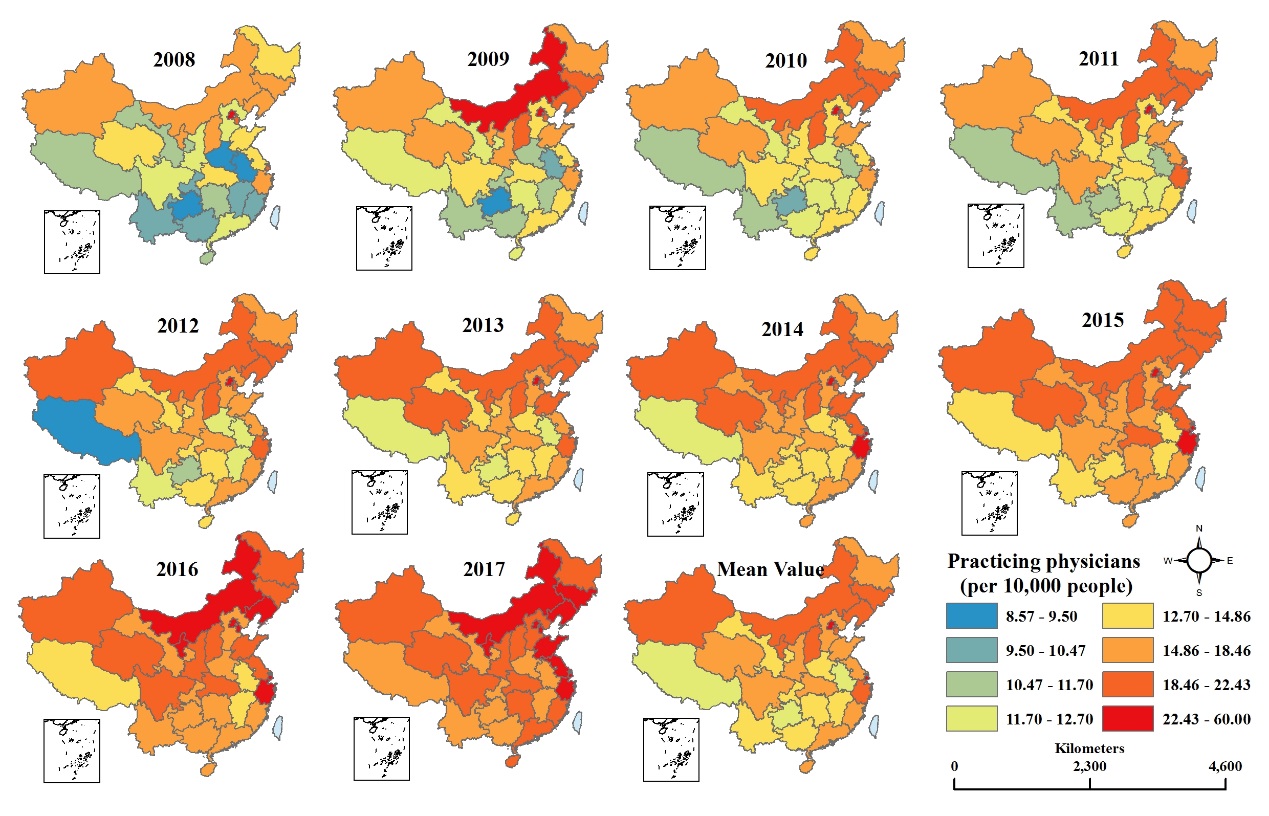


Figure S2 Number of licensed physicians per 10,000 persons in 31 provincial regions in mainland China from 2008 to 2017 (Map generated with ArcGIS 10.3 by authors)


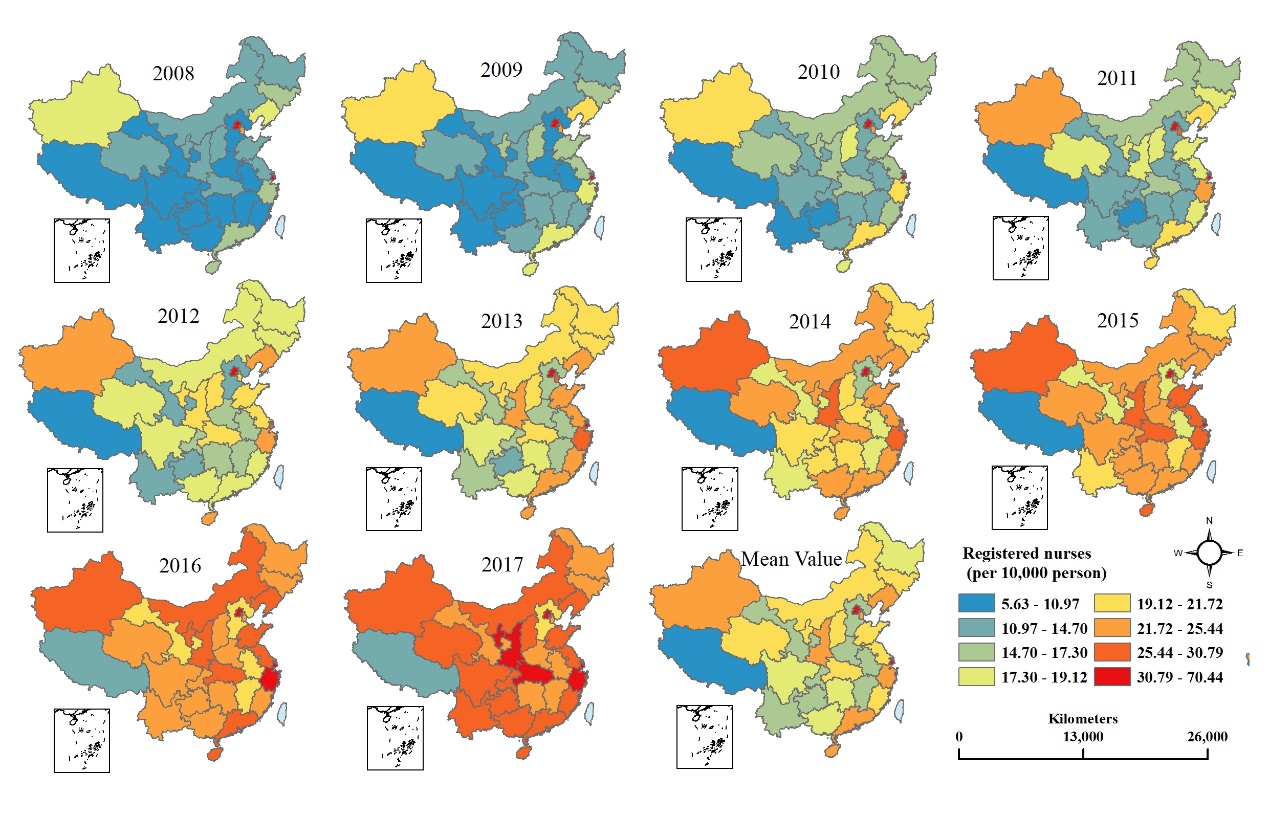


Figure S3 Number of registered nurses per 10,000 persons in 31 provincial regions in mainland China from 2008 to 2017 (Map generated with ArcGIS 10.3 by authors)


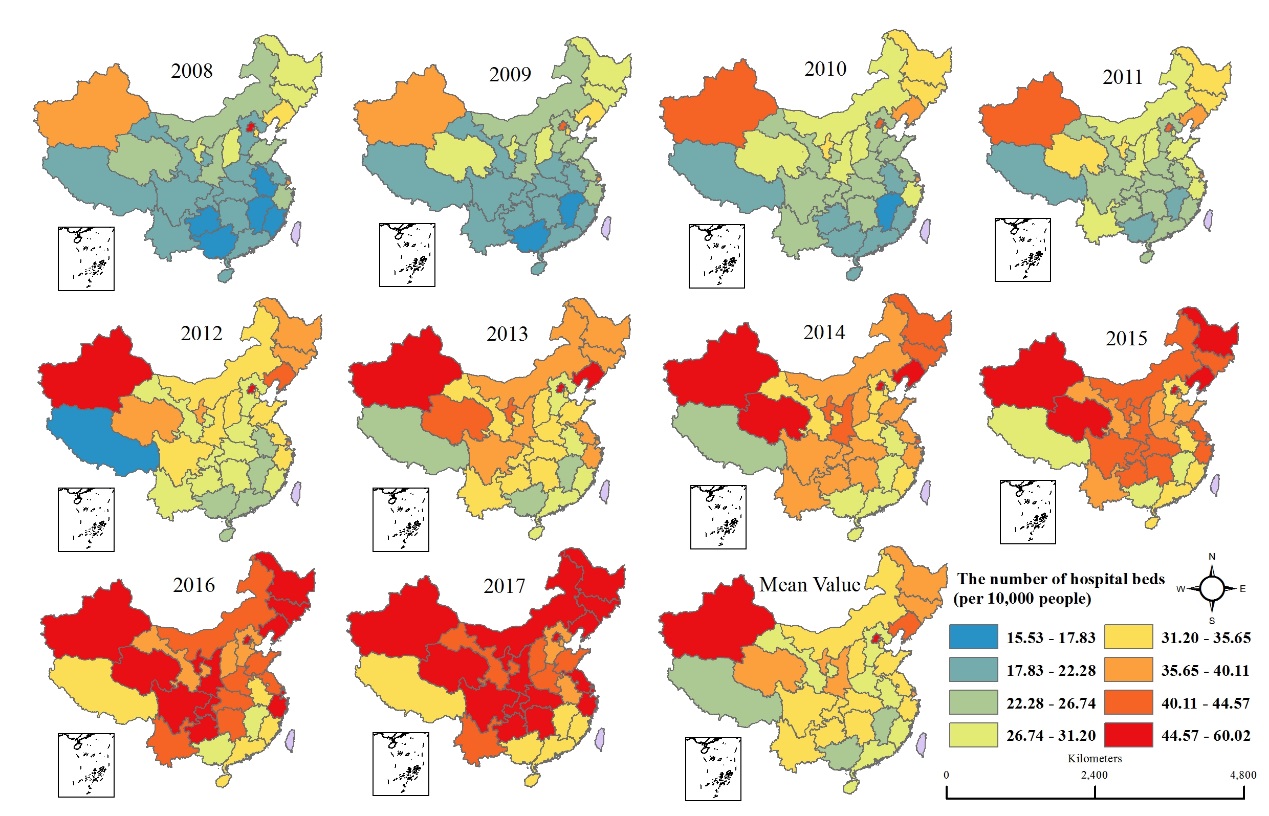


Figure S4 Number of beds in hospital per 10,000 persons in 31 provincial regions in mainland China from 2008 to 2017 (Map generated with ArcGIS 10.3 by authors)
